# Leadership in Nursing and Health Care in the Light of Complexity Thinking

**DOI:** 10.1590/1980-220X-REEUSP-2021-0553en

**Published:** 2022-05-30

**Authors:** Dirce Stein Backes, Regina Celia de Castro Gomes, Irani Rupolo, Andreas Büscher, Maria Júlia Pais da Silva, Carla Lizandra de Lima Ferreira

**Affiliations:** 1 Universidade Franciscana, Santa Maria, RS, Brazil.; 2 Hochschule Osnabrück, Osnabrück, Germany.; 3 Universidade de São Paulo, São Paulo, SP, Brazil.

**Keywords:** Nursing, Nursing Care, Nurse’s Role, Health, Leadership, Nonlinear Dynamics, Enfermería, Atención de Enfermería, Rol de la Enfermera, Salud, Liderazgo, Dinámica no Lineales, Enfermagem, Cuidados de Enfermagem, Papel do Profissional de Enfermagem, Saúde, Liderança, Dinâmica não linear

## Abstract

This study aims to perform a critical-reflective analysis of leadership in nursing and health in the light of complexity thinking. This is a theoretical-reflexive essay based on the framework of complexity thinking. A parallel is conceived between traditional Cartesian leadership, outlined from a defined linear hierarchical structure, and leadership from a systemic-complex perspective. A schematic structure is demonstrated between the pyramidal conception and systemic-complex leadership, leading to circularity, complementarity, interdependence, and interactivity. Leadership’s central role in nursing and health care is reaffirmed based on interactive, dialogic, and interdependent movements. The theoretical reflection calls for evolutionary and shared leadership in nursing and health, determined by the quality of interactions between members and different systems, in order to respond to the complexity of health phenomena quickly, effectively, and sustainably.

## RESUMO

Objetiva-se realizar análise crítico-reflexiva sobre a liderança em enfermagem e saúde, à luz do pensamento da complexidade. Ensaio teórico-reflexivo alicerçado no referencial do pensamento da complexidade. Concebe-se um paralelo entre a liderança tradicional cartesiana, esboçada a partir de uma estrutura hierárquica linear definida e a liderança na perspectiva sistêmico-complexa. Demonstra-se uma estrutura esquemática entre a concepção piramidal e a liderança sistêmico-complexa que conduz à circularidade, complementaridade, interdependência e interatividade. Reafirma-se o papel central da liderança em enfermagem e saúde, a partir de movimentos interativos, dialógicos e interdependentes. A reflexão teórica convoca à liderança evolutiva e compartilhada em enfermagem e saúde, determinada pela qualidade das interações entre os membros e os diferentes sistemas, no sentido de responder à complexidade dos fenômenos de saúde de forma rápida, eficaz e sustentável.

## RESUMEN

El objetivo es realizar un análisis crítico-reflexivo del liderazgo de enfermería y salud a la luz del pensamiento de la complejidad. Este es un ensayo teórico-reflexivo basado en el referencial del pensamiento de la complejidad. Se establece un paralelismo entre el liderazgo tradicional cartesiano, esbozado desde una estructura jerárquica lineal definida y el liderazgo, desde una perspectiva sistémica-compleja. Se demuestra una estructura esquemática entre la concepción piramidal y el liderazgo sistémico-complejo que conduce a la circularidad, la complementariedad, la interdependencia y la interactividad. Se reafirma el papel central del liderazgo en la enfermería y la salud, desde movimientos interactivos, dialógicos e interdependientes. La reflexión teórica reclama un liderazgo evolutivo y compartido en la enfermería y la salud, determinado por la calidad de las interacciones entre los miembros y los diferentes sistemas para responder a la complejidad de los fenómenos sanitarios de forma rápida, eficaz y sostenible.

## INTRODUCTION

Leadership understood as a constructive and disruptive process, has assumed, over time, different conceptions and meanings. In the period of modern capitalism, the references of leadership were the army, the clergy, and the fiefdom, with a predominance of coercive relationships. With the industrial revolution, mercenary leadership was accentuated, focusing on profit maximization at any cost. Finally, with the technical- scientific advance, a new leadership style has emerged today, with characteristics of greater centrality and interactivity among the followers^(1,2,3,4)^.

Beyond these notions and historical constructions, leadership can/should be conceived as an inductive tool for the (re)novation and evolution of the social system. This process is not about a great leader but leaders capable of connecting people and (re)creating effective, lasting, and sustainable solutions. Therefore, the leader is understood as a person, and leadership is a shared function between leaders and followers who assume, reciprocally and collaboratively, the evolutionary dynamics of a common cause^(5,6,7,8)^.

Leadership has gone through different theories and paradigmatic constructions in this evolutionary and disruptive movement. Nowadays, leadership is being constructed from a systemic-complex perspective, that is, in the direction of interactive and complementary thinking, in which the leader and the follower are not overlapped but exercise shared and interdependent functions. Complex thinking is characterized, under this view, by learning phenomena that emerge from a collection of interacting objects. As a circular and systemic phenomenon, leadership dynamizes the system’s evolution by understanding the part in the whole and vice-versa^(9,10)^.

Scholars, especially in the international sphere, have corroborated the need to consider new references for leadership support^(10,11,12,13)^. This systemic-complex perspective transcends the Cartesian approach to intervention, in which the leader held power and sought to get the most out of his subordinates, based on a defined linear hierarchical structure. Instead of a one-sided governance flow emphasizing linearity and predictability, a circular and synergetic movement of sharing ideas, experiences, and practices is created.

The health sector, a complex system, requires professional leaders who are willing to operate with unpredictability, ambiguities, and increasing complexity. In this context, the desired leadership must be systemically guided in order to manage processes with agility, safety, and quality^(14)^. In the Brazilian Unified Health System (SUS), built on the systemic-complex conception, leadership constitutes a driving force to catalyze different points of the health care network. Moreover, in this SUS system, as well as in global health systems, Nurses assume strategic and prospective leadership to achieve better health levels and reach the Sustainable Development Goals^(15)^.

From this point of view, the question is: Are nurses able to exercise circular, complementary, interdependent, and interactive leadership in Nursing and health? It is argued that in addition to increasing the number of nurses in leadership positions – a goal of the Nursing Now^(16,17)^ campaign – it is essential to weave systemic-complex references in teaching, research, and practice to strengthen the prospective and entrepreneurial leadership, aiming at the health systems dynamization.

Aiming to contribute to the evolution of systemic-complex thinking in the educational process and the nursing leaders’ qualification, assessing the competencies and skills necessary for the dynamization of change processes with agility, safety, and quality^(18)^, the present study aims to perform a critical- reflective analysis on nursing and health leadership in the light of complexity thinking.

## METHOD

This is a theoretical-reflexive study supported by the complexity-thinking referential. It analyzes the main elements that lead to leadership from a systemic-complex perspective. Morin, the author of complexity thinking, does not presume a predefined methodological path but rather encourages the researcher to understand that the urgency in the realm of ideas is not to review doctrines and methods but to develop a new conception of knowledge itself. It urges him, in this sense, to seek his own strategies based on the unpredictability, ambiguities, and complexities found in the real context^(9)^.

For the same author, traditional knowledge was subjected to a reductionist process that resulted in the loss of the multiplicity, diversity, and interdependence notions^(9)^. Under this approach, it enables a methodological itinerary in which the researcher, as the path’s protagonist, is induced to learn, invent, and autonomously (re)create his path, through interpretative processes in the here and now.

The present study is conducted from the systemic-complex thinking, which materializes in inventing, questioning, and weaving together the lived experiences in learning, teaching, investigating, leading, and caring in health. For this purpose, a schematic parallel is presented between traditional Cartesian leadership, conceived from a defined linear hierarchical structure, and leadership from a systemic-complex perspective, woven in the light of complexity thinking. We also consider fragments of the Editorial “Nursing Leadership for the 21st Century”^(18)^.

Therefore, the theoretical foundation is constituted by the author’s primary productions of complexity thinking^(19–22)^ and international reference articles about systemic-complex leadership. In this path, concepts such as circularity, complementarity, interdependence, and interactivity are explored without considering them conclusive or indisputable.

### From Pyramidal Conception to Systemic-Complex Leadership

To recover how complex social phenomena and human activities are, the author of complexity thinking^(21,22)^ induces a critical knowledge about thinking itself and its methods. This enables a continuous return to the dialogue with other ways of thinking. It is not a linear sequence but a spiral path that expands knowledge with each return to the understanding that learning occurs throughout life.

**Figure 1.**
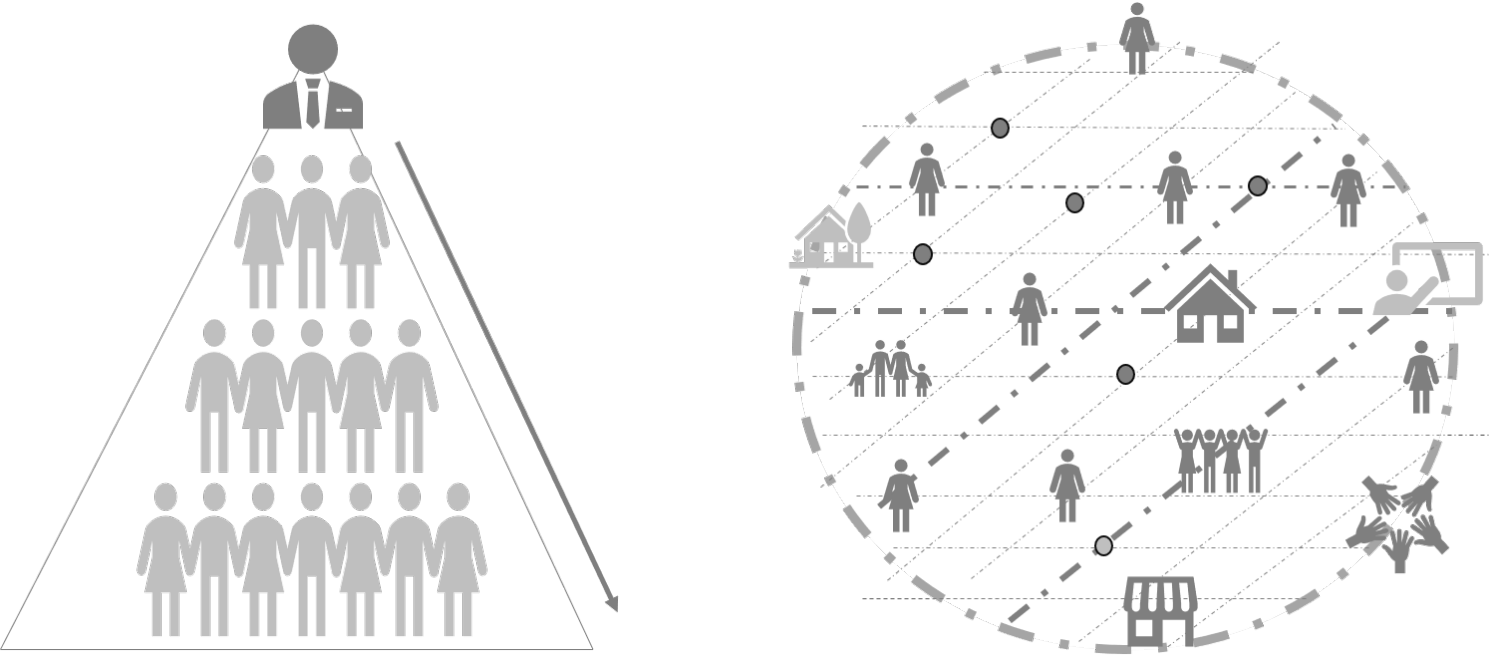
Pyramid structure and systemic-complex approach to leadership. Santa Maria, RS, Brazil, 2021.

Next, a schematic parallel between the pyramid structure and the complex-systemic approach is presented, to unveil evolutionary possibilities in terms of leadership. The predominant characteristics of each approach are shown, and it is suggested, not a rupture of the classical logic but an evolutionary complement concerning leadership, breaking with the reality’s complexity, as shown in Figure 1.

Based on the schematic parallel proposed, the theoretical- reflexive description is conducted by delineating two analysis categories: Pyramidal structure: defined, linear, and point functions; and Systemic-complex leadership: from defined linearity to interdependence.

### Pyramidal Leadership: Defined, Linear and Punctual Roles

The pyramid structure has as its predominant characteristics the fractioned functioning in sectors and specialties, order, and a centralized decision-making process at the top of the organization. In this logic, there is a predominance of vertical interaction between superiors and subordinates, rigid control systems, and greater reliance on formal rules and protocols. The employees participate little, and their ideas and suggestions rarely reach the top management. In this relationship, employees generally work under a unilateral, compartmentalized order. In this logic, the tendency is for the results to be the same.

Most of the time, the chief leader assumes authoritarian characteristics, determined by power, which over time erode relationships and hostilize the work environment. Technical and punctual knowledge about human interactions also prevails, and there is more interest in maintaining power than in the employees and the organization’s objectives. The position, in many cases, is above any interest.

The traditional pyramid structure offers an advantage for local short-term decisions since the boss, in his hierarchical function, can direct and control the workflow in a faster, more effective, and controlled way. This structure demands middle management to delegate tasks and convey the most qualified leaders’ business vision. In this structure, however, the employees’ skills, talents, and ideas at the pyramid’s base (lower level) are rarely perceived and leveraged since there is more emphasis on compliance with norms and routines. This routine and the automated process can easily lead to the employee’s demotivation and sickness, as well as contribute to the dehumanization of health care.

The pyramidal, fractioned, and specialized conception has become, under this approach, insufficient to respond to the growing healthcare complexity. In addition to Head Nurses, it is necessary to have leaders prepared to make collegiate and interprofessional decisions that can value and enhance individual and collective talents. The demand for effective leaders in nursing is decidedly urgent and necessary, according to the Editorial “Nursing Leadership for the 21st Century”^(18)^:

Promoting “great Clinical Nurses” into management is no longer a viable model. Success for healthcare organizations in meeting fiscal, patient satisfaction, and quality of care goals depends primarily on the Nursing leadership competencies^(18)^.

There must be a paradigm shift in the education and mentoring of Nursing leaders, with a radical reevaluation of the qualities and skills necessary for Nursing leaders to move Nursing forward into the 22nd century and beyond. Change is necessary as the rapid transformations we are witnessing in the healthcare environment will continue. Leaders must be appropriate to drive a rapid change process plan to ensure success effectively^(18)^.

Thus, there is an urgent need to advance in the direction of systemic-complex thinking. Although it is comfortable to maintain the order, organization, and logic stability that has been in place for years, it is indispensable to keep up with the evolutionary dynamics of systems, which has become even more acute with the Covid-19 pandemic. For the complex thinking author, a strictly deterministic system, which consists only of order, would be a universe without becoming, without innovation, and without creation^(22)^. However, how can we overcome the deterministic pyramidal structure in order to develop circular, complementary, interdependent, and interactive leadership in nursing and health care?

### Systemic-Complex Leadership: From Defined Linearity to Interdependence

Understanding a system is related to a mutually interacting complex of elements. This representation can be applied to the person, family, organizations, and society. Each system can be subsidized by subsystems and be inserted into other larger systems^(19,20)^. Thus, in a systemic organization, such as SUS, users, services, and leadership are interdependent and components of a system that is the organization itself. Change in a subsystem is (re)produced according to its own dynamics and interactions with the larger system, which is also changeable.

In the systemic-complex approach to leadership, organizational arrangements are flexible, decentralized, and interconnected. Roles are (re)defined by the interaction between the people who perform the different activities. Leadership, located at any point in the network, becomes dynamic based on multidimensional knowledge. In this logic, horizontal interaction predominates over vertical interaction. Leadership success or failure is determined by the quality of the interactions among members and subsystems, by the ability to build prospective relationships, and by adopting collective, long-lasting, and sustainable strategic actions, as shown in the following fragments:

Nursing leaders must be able to create and communicate a vision for their areas of responsibility. They need to build positive relationships with those they lead and adopt plans and actions to achieve mutual goals in order to be successful. Communication must be frequent and ongoing, with a two- way dialogue^(18)^.

There must be a call to action to ensure that leaders in Nursing at all management levels have not only the skills and experience to move and lead organizations. These leaders must have a seat at the table. Leaders in Nursing need to be able not only to create and participate in decisions in health policy. They must also be able to manage the healthcare team members throughout the healthcare organization^(18)^.

To lead, in the systemic-complex logic, implies understanding that any change in one of the system’s parts simultaneously affects the whole; that the whole is in the parts and the parts in the whole^(22)^; that it is necessary to live with differences, dialogue in adversity, negotiate amidst uncertainties, and continuously reinvent oneself. It also implies the ability to integrate the notions of order and disorder; t deal with conflicts; readjust to the changing environmental conditions, as well as to receive and provide qualified interactions to multiply itself creatively and prospectively.

In contrast to mechanistic metaphors that emphasize a mode of control and stability, systemic-complex leadership uses the metaphor of a living organism to demonstrate disruptive evolution^(10)^. The post-Covid-19 society will be essentially dynamic and changeable. Therefore, to keep up with the environment and the effervescent moving systems, the organizations/systems/professions, in this case, Nursing should be highly organic, in other words, innovative, temporal, and anti-bureaucratic^(23)^.

### Nursing Leadership from a Systemic-Complex Perspective

From the systemic-complex leadership perspective, we discuss the conceptions of circularity, complementarity, interdependence, and interactivity, which are in dialogue with the three principles proposed by the complexity thinking author^(21,22)^, namely: the dialogic, the organizational resource, and the hologramatic. For the mentioned author, systems theory is broad and all known reality can be conceived as a system. For the author, it is the interactions that modify the behavior and the phenomena, which determine the evolutionary dynamics of a system, whatever it may be.

In the dialogic principle, the complexity thinking author conceives the integration of ideas that are, simultaneously, complementary, competing, and antagonistic, so that the conflict between the opposites is always the impulse for the system’s renewal. Dialogic does not break with the antagonisms between the parts/subsystems but considers and integrates them constituting a symbiosis, indispensable to the imminence of a new social order or a more advanced knowledge level. It is important, therefore, that a system is open to the influences of the external environment and not self-enclosed. A system closed to dialogic does not reorganize itself and, therefore, does not innovate or evolve^(20–22)^. Systemic-complex leadership requires, under this impulse, (retro)feeding from external elements and the interactions with its constituents, in other words, among its followers, subsystems, and professional areas.

Unlike pyramidal logic, which considers a single reality level, the systemic conception holds multiple realities and moves in various directions to form the complex unit. In this sense, dialogic allows the integration of ideas, to enable a third possibility, even broader and more complex^(21,22)^. Thus, based on the dialogic principle, systemic-complex leadership will be contributing to the evolution and continuous innovation of the system.

In the organizational resource principle, in which products and effects are, at the same time, the cause that produces them, the complex thinking author defends the interactivity between systems and subsystems. The expressions self-organization and eco-organization seek to express the inseparable relationship between subject and world since the world is in the subject just as the subject is in the world. Self-organization constitutes eco-organization, which, in turn, integrates self-organization in a recursive, recurrent, and complex relationship^(19–22)^. In this direction, it is necessary to treat as antagonistic and complementary the one and the diverse, the whole and the parts, order, and disorder, interprofessionalism, inter-sectoriality, and the different systems and social actors that interact in/with leadership relationships.

Under this impetus, there is a pressing need for systems (re)organization, mobilized by the movements of dialogic that favor the conditions for the (re)organization of teaching and learning, research, and practice. All organizational change brings along disorder, noise, and deviation. Although antagonistic, these terms are complementary and interconnected and, therefore, indispensable when proposing the regeneration and evolution of an organization^(22)^.

Systemic-complex leadership is driven to build an organization that maintains defined functions, that is open to tensions and regenerations, and that admits, concomitantly, the disorder and disturbances of the environment^(5)^. Organizational renewal is, then, the outcome of a leadership process that can conceive and deal with everyday adversities and randomness.

In the hologrammatic principle, the complexity author conceives the whole as being greater than the sum of the parts. In this relationship, the whole is in the part, just as the part is in the whole. This idea transcends reductionism which sees only parts/subsystems or holism which sees only the whole/system^(20,21)^. Systemic-complex leadership develops, under this approach, from the notion of whole and part, both in mutual interaction, and success is deliberated by the quality of interactions. This means understanding that effective leadership enables synergistic interactions with other systems, services, and professionals.

In this systemic-complex relationship, leadership is constituted as a collaborative process in which formally designated individuals can play the role of leader in constant synergy with the followers. The leader is invited to promote inclusion mechanisms of the actors present in leadership, acting in it, influencing it, and continuously (re)creating it^(7,10)^. Thus, leadership is represented collectively and collaboratively in the situation and as a consequence of the relationships and interactions of its actors.

Leadership, in the complex-systemic perspective, is simultaneously woven of circularity, complementarity, interdependence, and interactivity. From this perspective, nursing’s central role in the health system is reaffirmed, given the unpredictability, uncertainties, and ambiguities of reality, which implies the ability to transcend verticalized paradigms and establish prospective connections between the different network points^(23,24)^. To act in the SUS and cope with the complexity of the social system, the nurse needs to increasingly develop skills and competencies in teaching, research, and practice, aware of the need for lifelong learning.

Therefore, this new paradigm can contribute to the (re)construction of new, more relevant, and useful knowledge, in other words, knowledge that can help in the (re)formulation of new development proposals that are more flexible, critical, and able to self-(re)organize. Developing a new way of thinking about leadership, in the light of systemic-complete thinking, is fundamental to find interactive possibilities that add value and impact both society and the health system.

However, paradigmatic change is a complex process that generates the deconstruction of the culturally established structure of ideas. It is associated with the academic world, in the way scientific knowledge is constructed, investigated, and managed. Change implies a distinct look at the living world experiences, in the sense of provoking ruptures and conceiving new methodological and research possibilities.

Although the theoretical reflection has been woven in the light of complexity thinking, practical aspects of leadership can/should gradually incorporate different theories in a broader and more complex construction. The future challenge is to reconcile, from a (re)constructive and disruptive dynamic, the combination of different theories related to leadership, in order to provoke innovations without incurring new ruptures and reductionisms.

This study’s contributions to the advancement of scientific knowledge are associated with the induction of nursing and health leadership, generating evolutionary thinking without, however, provoking new reductionisms. Under this evolutionary thinking, there is a need to overcome punctual and linear practices, supplant vertical and dichotomous models of intervention, as well as to dynamize agile, resolutive, and sustainable processes. The Brazilian Health System is, by excellence, dynamic, associative, and articulated in networks and lines of care. As a result, leadership will be a skill that is increasingly esteemed and stimulated in the different professional areas.

This study is limited by the reduced number of national publications on leadership from a systemic-complex perspective, as well as by the challenge of designing new references in leadership without causing new reductionism. We propose that the questions posed in this study raise new reflections and corroborate complex thinking, which leads to the interconnection of different knowledge, services, and health sectors, aiming at practice innovation.

## CONCLUSION

This theoretical reflection calls for evolutionary and shared leadership in Nursing, determined by the quality of interactions between members and different systems, in order to respond to the complexity of health phenomena in a rapid, effective, and sustainable manner. From a theoretical-reflexive parallel between the pyramidal structure and the systemic-complex leadership, it reaffirms the central leadership role in the innovation of Nursing and Health practice.

Beyond certainties and absolute truths, this study raises the relevance of evolutionary, prospective, and horizontal leadership, without fearing interprofessional conflicts, disorder, or chaos necessary for evolution.

Complex-systemic thinking is relevant because it prioritizes relationships, interactions, and systemic associations to address and propose prospective leadership strategies. In short, it advocates a set of theoretical reflections elaborated from multiple dimensions, in order to provoke innovations without incurring new reductionisms.

## ASSOCIATE EDITOR

Paulino Artur Ferreira de Sousa
